# Supplementary material for: Low-noise frequency-agile photonic integrated lasers for coherent ranging
Source: Nat Commun. 2022 Jun 20;13:3522. doi: 10.1038/s41467-022-30911-6 (PMC9209488; doi:10.1038/s41467-022-30911-6)
Supplement: Supplementary file 1 — Supplementary Information [file 41467_2022_30911_MOESM1_ESM.pdf]

o

Supplementary Information for Low-noise frequency-agile photonic integrated lasers for coherent ranging

Grigory Lihachev,[1,*] Johann Riemensberger,[1,*] Wenle Weng,[1,*] Junqiu Liu,[1] Hao Tian,[2] Anat Siddharth,[1] Viacheslav Snigirev,[1] Vladimir Shadymov,[1] Andrey Voloshin,[1] Rui Ning Wang,[1] Jijun He,[1] Sunil A. Bhave,[2,†] and Tobias J. Kippenberg[1,‡]

[1]*Institute of Physics, Swiss Federal Institute of Technology Lausanne (EPFL), CH-1015 Lausanne, Switzerland*
[2]*OxideMEMS Lab, Purdue University, 47907 West Lafayette, IN, USA*

Simulation of the laser self-injection locking dynamics

The conventional laser rate equation [1] for describing the semiconductor laser field dynamics can be written as:

$$\frac{dA_{\mathrm{laser}}}{dt} = \frac{1}{2}(1 - i\alpha_{\mathrm{H}})[aV(N - N_0) - \frac{1}{\tau_{\mathrm{p}}}]A_{\mathrm{laser}} + F_{\mathrm{A}} \tag{1}$$

where A_{laser} is the complex laser field profile and $|A_{\mathrm{laser}}|^2$ is the averaged photon density in the laser cavity, N is the carrier density, α_{H} is the linewidth enhancement factor, a is the differential gain, V is the laser active volume, N_0 is the carrier density at transparency, and F_{A} is the Langevin noise term to describe the spontaneous emission into the lasing field [2]. This Gaussian noise term obeys $\langle F_{\mathrm{A}}(t)F^*_{\mathrm{A}}(t')\rangle = 4\beta N B_{\mathrm{sim}}\delta(t - t')$. Here β is a factor related to the amount of spontaneous emission into the lasing mode, B_{sim} is the simulation bandwidth. We note that the noise in the carrier density fluctuation is ignored in our simulations because its contribution to the laser noise is insignificant in comparison to the noise in the optical field [3].

Symbol	Value	Unit	Definition
α_{H}	5		Linewidth enhancement factor
a	1×10^4	s^{-1}	Differential gain
N_0	1×10^{24}	m^{-3}	Carrier density at transparency
κ	$1 \times 10^{11} \times 2\pi$	rad s^{-1}	Laser cavity loss rate
I_{bias}	250	mA	Biased current
ζ	5×10^8	Hz/mA	Current-frequency tuning coefficient
γ	1×10^9	s^{-1}	Carrier recombination rate
V	2×10^{-16}	m^3	Volume of active section
β	1×10^4	s^{-1}	Spontaneous emission coefficient
e	1.6×10^{-19}	C	Elementary electronic charge
κ_{r}	$60 \times 10^6 \times 2\pi$	rad s^{-1}	Loaded loss rate
κ_{ex}	$50 \times 10^6 \times 2\pi$	rad s^{-1}	External coupling rate
κ_{sc}	$30 \times 10^6 \times 2\pi$	rad s^{-1}	CW-CCW coupling rate
κ_{inj}	$7.5 \times 10^{16} \times (2\pi)^2$	rad^2 s^{-2}	Laser-microresonator coupling factor
ϕ	1.5	rad	Feedback phase
n_2	2.4×10^{-19}	m^2/W	Kerr nonlinear index
n_0	2		Microresonator refractive index

Table I. Values and definitions of parameters used in the simulations.

We incorporate the laser rate equations with the equations for the coupled fields in a clockwise (CW) mode (which is the mode directly pumped by the laser field) and the counter-clockwise (CCW) mode in the microresonator to numerically study the self-injection locking dynamics. We also add a phenomenological term in the laser field equation in order to include the current-frequency tuning ability with a coefficient of 500 MHz/mA. This frequency tuning ability originates from several mechanisms include gain compression, carrier-density-dependent refractive index change and temperature change [3, 4]. Since only ~ 10 mA is applied for the current modulation amplitude, the linear approximation agrees well with the experimental observations. The coupled equations are written as:

$$\frac{dN}{dt} = \frac{I_{\mathrm{bias}} + \Delta I}{eV} - \gamma N - aV(N - N_0)|A_{\mathrm{laser}}|^2 \tag{2}$$

$$\frac{dA_{\text{laser}}}{dt} = \left[\frac{1}{2}(1 - i\alpha_{\text{H}})(aV(N - N_0) - \kappa) - i\zeta\Delta I\right] A_{\text{laser}} + \sqrt{\kappa_{\text{inj}}} e^{i\phi} A_{\text{CCW}} + F_{\text{A}} \tag{3}$$

$$\frac{dA_{\text{CW}}}{dt} = (-\kappa_{\text{r}} + i\delta\omega(t) + i\Gamma(|A_{\text{CW}}|^2 + 2|A_{\text{CCW}}|^2)A_{\text{CW}} + i\kappa_{\text{sc}} A_{\text{CCW}} + \sqrt{\kappa_{\text{inj}}} e^{i\phi} A_{\text{laser}} \tag{4}$$

$$\frac{dA_{\text{CCW}}}{dt} = (-\kappa_{\text{r}} + i\delta\omega(t) + i\Gamma(|A_{\text{CCW}}|^2 + 2|A_{\text{CW}}|^2)A_{\text{CCW}} + i\kappa_{\text{sc}}^* A_{\text{CW}} \tag{5}$$

Here the carrier recombination rate is represented by γ, the elementary charge is denoted by e, $|A_{\text{CW}}|^2$ and $|A_{\text{CCW}}|^2$ are the averaged photon densities in the CW microresonator mode and the CCW microresonator mode respectively, κ_{r} is the microresonator decay rate, κ_{sc} is the coupling rate between the CW and the CCW modes, κ_{inj} is the coupling rate between the microresonator and the laser, and ϕ is the coupling phase related to the gap between the laser and the microresonator. The Kerr frequency shift coefficient can be calculated using $\Gamma = \frac{\hbar\omega_0^2 c n_2}{n_0^2}$, where n_0 is the refractive index of the microresonator material and n_2 is the Kerr nonlinear coefficient. The detailed definitions and values of the parameters used in the simulations are presented in Table I.

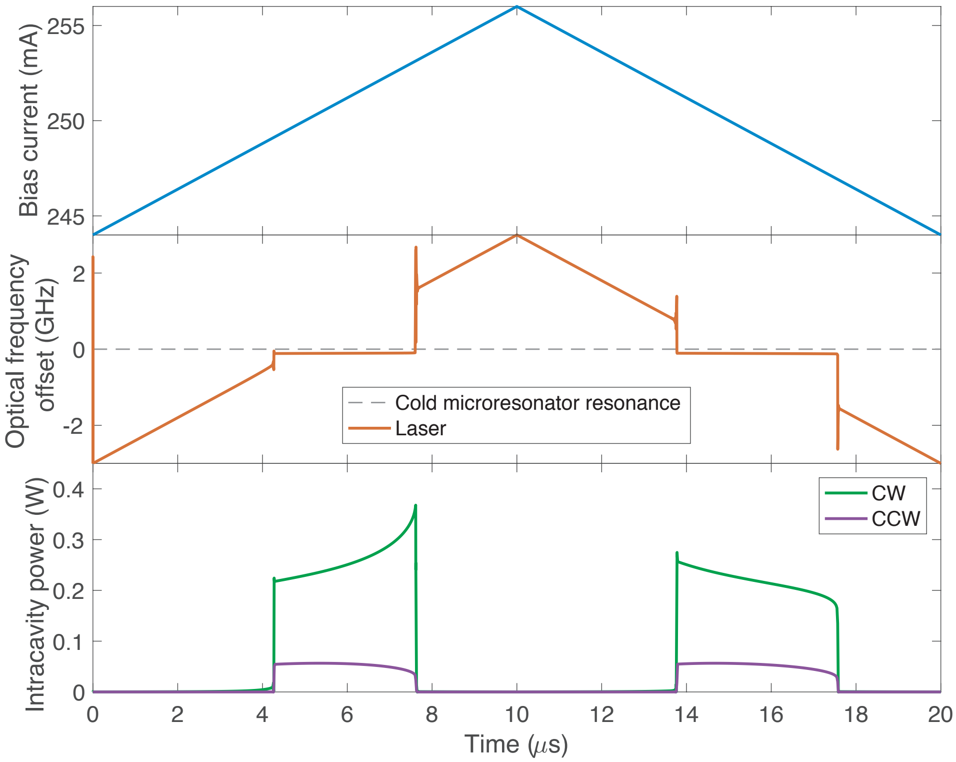

Figure 1. **Simulated self-injection locking with laser current tuning.** The bias current is tuned forward and backward with a total tuning range of 12 mA, corresponding to a natural lasing frequency range of 6 GHz. The middle panel presents the frequency shift of the laser, showing qualitative agreement with the experimental result in Fig. 1 (c) in the main text. In the injection-locked ranges, the small frequency gap between the cold microresonator resonance and the lasing frequency is caused by the Kerr-effect-induced frequency shift of the microresonator resonances. In the lower panel, the intracavity powers of the CW and CCW fields are plotted.

Fig. 1 shows the simulation results when the laser bias current is swept over 12 mA. The laser frequency shows the self-injection-locking phenomenon at both current-up and -down sweeping directions. We adjust the feedback phase ϕ, so the locking ranges with different sweeping directions are similar. Prior studies show that a well-selected phase facilitates the maximization of the locking range [5]. The simulated locking range of a few gigahertz is in good agreement with the experimental results presented in Fig. 1 (c) in the main text.

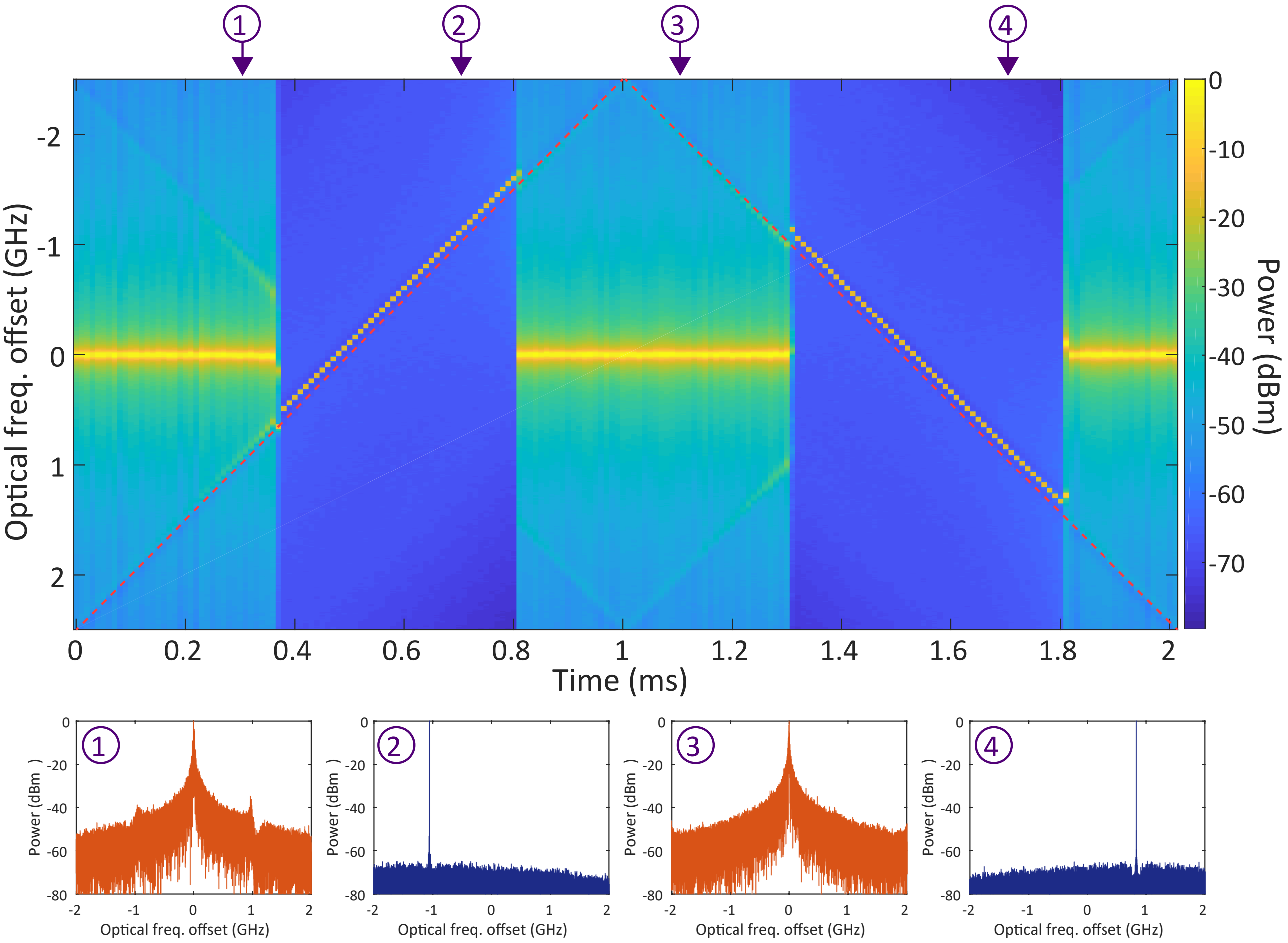

Figure 2. **Simulated laser frequency spectrogram as the microresonator resonance is swept back and forth.** Each frame in the spectrogram is normalised, so the peak power is 0 dBm. The red dashed lines show the cold microresonator resonance frequencies. Four spectra labeled with 1 – 4 are shown at the bottom, corresponding to the numbered positions indicated in the spectrogram. The spectra of the self-injection-locked laser (in blue) show significantly narrower lasing linewidth and lower noise floor than those of the free-running laser (in red).

Next, we sweep the cold microresonator resonance frequency over the semiconductor laser's natural lasing frequency with both frequency-up and frequency-down directions while the laser bias current is kept constant. We use fast Fourier transform to compute the laser optical spectra at varied laser-microresonator detunings. Fig. 2 shows the full spectrogram. The evolution of the lasing frequency is similar to the results in [5]. Additionally, here with the inclusion of the spontaneous emission noise in our simulations, the significant improvement of the laser noise performance of the self-injection locking state is clearly shown.

We compute the laser frequency noise power spectral density (PSD) with the simulation data to characterize the laser linewidth narrowing effect [6]. In Fig. 3 the PSDs of the free-running (without self-injection) laser and the self-injection-locked laser are compared. This comparison indicates that within the resonance bandwidth of the microresonator the linewidth narrowing factor is larger than 40 dB, showing excellent agreement with the experimental results presented in Fig. 2 (d) in the main text.

To investigate the frequency modulation bandwidth of the hybrid laser, we fix the laser bias current and modulate the cold microresonator resonances with a sine function whose modulation amplitude is of 1 GHz. The modulation frequency (f_{mod}) is varied from 100 kHz to 50 GHz. The simulated responses of the self-injection-locked laser frequency are displayed in Fig. 4 (a) and (b), showing that the laser frequency response is not limited by the resonance bandwidth of the microresonator that provides the feedback for self-injection locking. This result may seem counterintuitive at first. However, our simulations show that when the resonance-modulated microresonator is pumped by a free-running laser, the strong resonance modulation will produce sidebands in the intracavity fields, even when the modulation frequency is much higher than the resonance bandwidth (see Fig. 4 (c) and (d)). The created sidebands are fed back to the laser, introducing the amplification of the sidebands. As a result, as long as the sidebands are within the

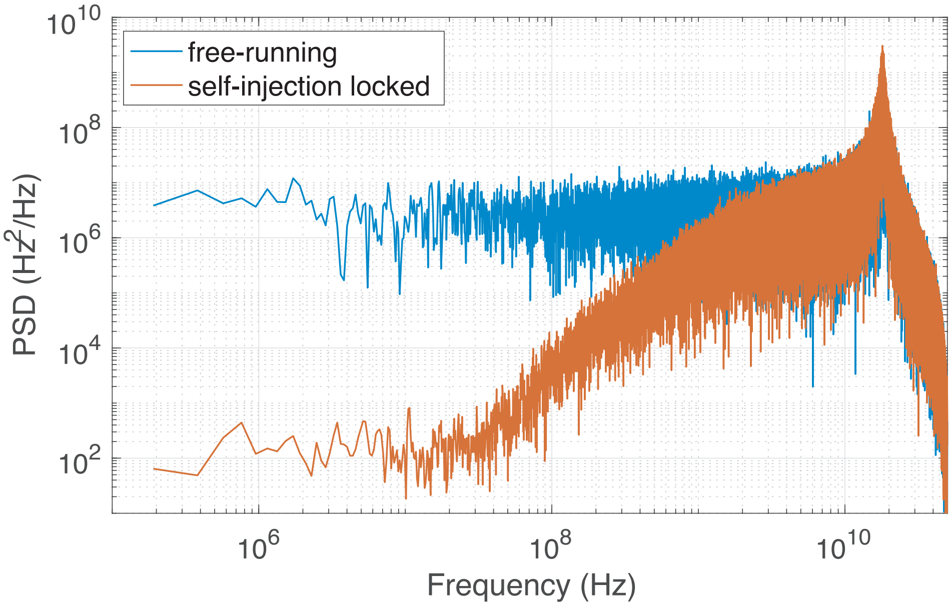

Figure 3. **Simulated laser frequency PSDs.** Within the microresonator bandwidth (i. e., 60 MHz), the linewidth narrowing factor is more than 3×10^4. The peaks at ~ 20 GHz are the relaxation oscillations.

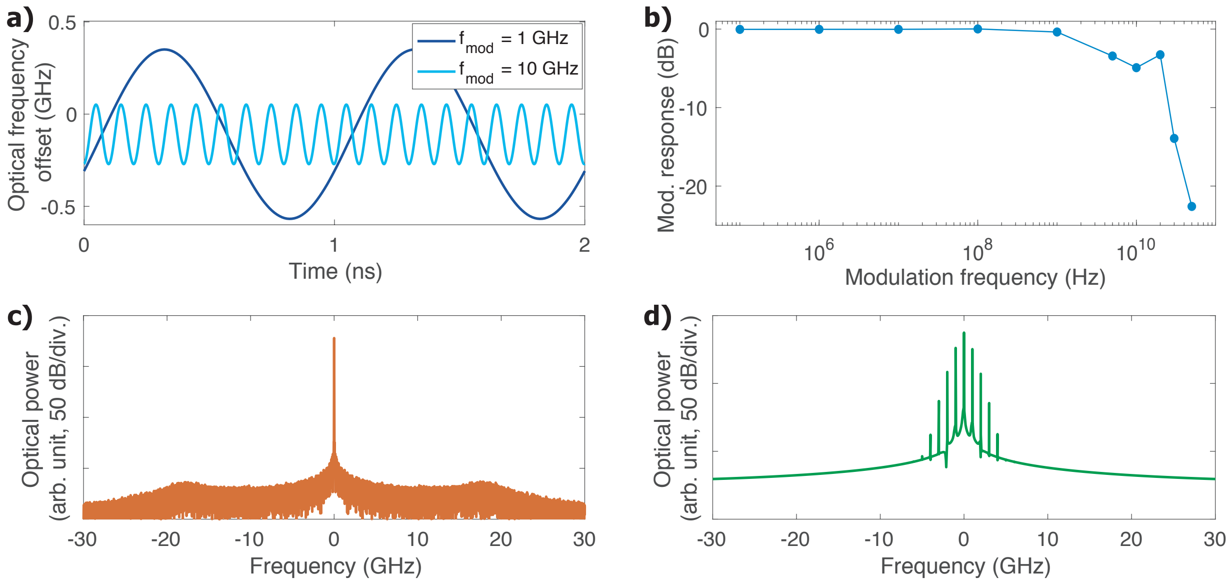

Figure 4. **Simulated microresonator-resonance-modulation responses of the self-injection-locked laser frequency.** **(a)** With the cold microresonator resonances being modulated with a modulation range of 1 GHz at two different modulation frequencies ($f_{\mathrm{mod}} = 1$ and 10 GHz) respectively, the self-injection-locked laser frequency responses are presented. **(b)** The transfer function of the modulation response. The rise at $f_{\mathrm{mod}} = 20$ GHz is due to the nearby relaxation oscillation frequency at 17.8 GHz. **(c)** Simulated optical spectrum of the free-running laser. **(d)** Simulated optical spectrum of the intracavity CW field when the microresonator is driven by the free-running laser and resonance-modulated with $f_{\mathrm{mod}} = 1$ GHz.

laser cavity resonance bandwidth, the laser may be effectively frequency-modulated while self-injection locked. Of course, in practice, the nonlinear response and the dispersion of the laser gain medium could have a negative impact on the feedback locking dynamics, and in reality, most of the time, the modulation actuation mechanism, i. e., the piezoelectric actuation in this work poses the bottleneck of the modulation bandwidth. Yet, our simulation shows the promise for ultrahigh modulation bandwidth for lasers that are self-injection locked with ultrahigh-Q microresonators.

Fabrication of photonic chips with integrated AlN piezoactuators

Microresonators and bus waveguides are fabricated with the photonic Damascene process [7], deep-ultraviolet stepper lithography[8] and silica preform reflow[9]. The waveguide cross-sections were optimized to minimize field overlap with the waveguide sidewalls and scattering loss. The bus waveguides have the same cross-section as the microring resonators and are terminated at the chip facets with 250 nm wide double-inverse tapers [10] both for out-coupling to optical fibre and interfacing with the DFB laser. The input coupling loss is 7 dB and can be improved by using dual layer Si_3N_4 inverse tapers to match the optical mode of a DFB laser. To fabricate the piezoelectrical actuator, 100 nm Mo and 1 μm polycrystalline AlN films are sputtered on SiO_2 cladding through foundry services (OEM Group). The AlN disk is first patterned with standard UV photolithography and dry-etched using Reactive Ion Etching (RIE) with chemicals Cl_2 and BCl_3. The dry etching of the bottom electrode (Mo) is performed using Cl_2. Finally, the top 100 nm of Al is evaporated and patterned using a standard lift-off process.

Supplementary figure 5 shows the frequency-dependent transmission and reflection of the photonic chips used throughout this work. We employ frequency comb-calibrated laser spectroscopy to perform linear characterization of photonic chips [11]. The fundamental TE mode is chosen in all cases for operation. The microresonators used in the phase noise measurements and the FMCW LiDAR experiment (main manuscript Figs. 2,3,4) have the following properties: The 2.45 GHz FSR device has 9300 μm radius, 800 nm waveguide core height, 900 nm waveguide core width , and loaded $Q=7.7\times10^6$. The 9.87 GHz FSR device has 2320 μm ring radius, 900 nm waveguide core height, 2100 nm waveguide core width, and loaded $Q=10.1\times10^6$. The 190.7 GHz FSR device has 120 μm ring radius, 950 nm waveguide core height, 1800 nm waveguide core width, and loaded $Q=4.8\times10^6$. The statistical distribution of waveguide loss rates is rooted in the weak interactions between the fundamental and higher order waveguide modes and their periodic mode crossings.

The FMCW LiDAR experiment is carried out using a microresonator with a free-spectral range (FSR) of 190.7 GHz. The reflection response of the photonic chip is composed of the backreflection of the chip facets and from the microresonator. The former induces sinusoidal modulations of the chip transmission and reflection. The latter leads to a narrowband reflection feature that interferes with the facet backreflection. For large resonators, we do not observe a mode splitting due to the intracavity backreflection. A typical Fano shape is observed as the two backreflection pathways interfere, modulating the on resonance response and limiting the injection locking bandwidth for many resonances. However, due to the very high Q of the resonators, even minuscule reflections are sufficient to achieve tight injection locking of the laser [12]. We also note that the wavelength-sized gap and the high refractive index of the InP laser diode will change the phase and amplitude of the sinusoidal modulations compared to the measurement with two lensed fibres.

Thermal management of the hybrid integrated laser

The AlN actuator does dissipate 240 nW at DC and 2.9 μW at 1 MHz. The DC value is based on our measurement of a 2 nA leakage current at 120V. The AC value is calculated from the equivalent series resistance (ESR) of the AlN dielectric film of a 112 μm disc actuator with thickness 1 μm and loss tangent 2.1×10^{-3}. We do not observe a significant red-shift of the microcavity resonance due to heating upon starting the fast AlN actuation. The thermal management of a packaged hybrid integrated system (the experiments were conducted on an electro-optical probe station) is estimated as follows: The InP laser chip dissipates around 1.5 W heat and would be mounted on a TEC with active temperature control. The photonic Si_3N_4chip would be mounted on a separate TEC for temperature stabilization. The AlN actuator hence will not contribute to the thermal budget of the final packaged device.

Frequency noise measurement

The laser frequency noise is measured using two methods: First, we perform heterodyne beat note spectroscopy using an external cavity diode reference laser (Toptica CTL), which we lock to a crystalline whispering gallery mode microresonator via the Pound-Drever-Hall (PDH) technique to suppress characteristic excess phase noise of the diode laser between at offset frequencies of up to 10 kHz. The noise of the reference laser is determined by heterodyne spectroscopy with a fibre laser locked to an ultra-stable optical cavity (MenloSystems ORNS) at 1553 nm while beeing locked to a different resonance of the same mode family of the crystalline resonator. Direct comparison of the ultrastable reference laser at 1552.5 nm is not possible due to a large wavelength offset of DFB, operating at 1556 nm at 26°C temperature. The electrical output of the photodiode is fed to a phase noise analyzer (Rhode & Schwarz FSW43). The phase noise of the lasers is determined via Welch's method from a 1 second time sampling trace (sampling rate 40 MHz) of the in-phase and quadrature components of the beat note and transformed according

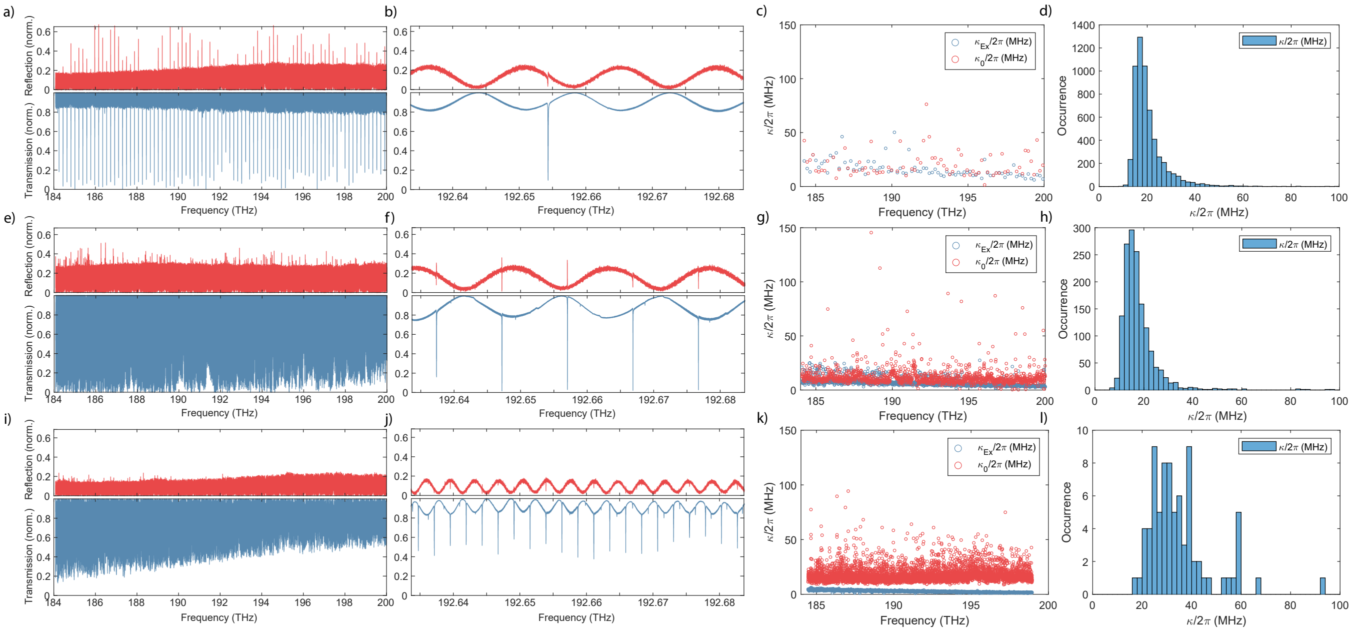

Figure 5. **Linear spectroscopy of photonic components** a,b) Transmission (blue) and reflection (red) of bus waveguide coupled to photonic Damascene microresonator with free-spectral range (FSR) 190.7 GHz (D45). c) Frequency-dependent microresontor loss $\kappa_0/2\pi$ (blue) and bus waveguide coupling $\kappa_{ex}/2\pi$ (red). d) Histogram of loaded microresonator loss rate $(\kappa_0 + \kappa_{ex})/2\pi$ rate. e-h) Same as a-d) but for 9.87 GHz FSR microresonator (D50). i-l) Same as a-d) but for 2.45 GHz FSR microresonator (D56).

to $S_{ff} = f^2 \cdot S_{\phi\phi}$ to yield the laser frequency noise. Fig. 6 shows the full plot for the frequency noise of the hybrid integrated laser highlighting the low-offset frequency sections down to a offset frequency of 10 Hz. The self-injection-

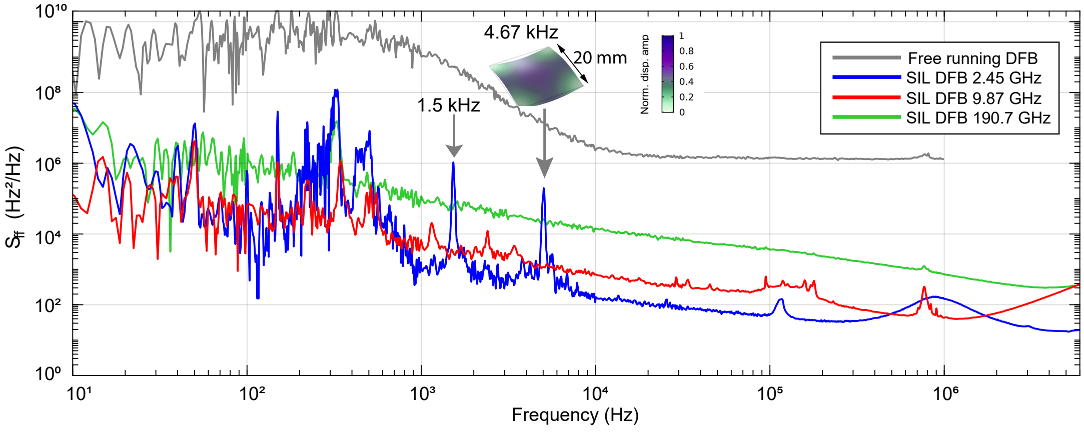

Figure 6. **Frequency noise of the hybrid integrated laser measured with the single reference laser.** The colors correspond to the colors in the main manuscript Fig. 2. We mainly attribute excess phase noise at 100–700 Hz to vibrations of the experimental setup. The peak at 1.5 kHz is the technical noise from a vacuum pump placed in the lab. We attribute the peak at 5 kHz to the excitation of the mechanical contour mode of the square 20 mm Si_3N_4chip. The peaks at 100-200 kHz are attributed to the reference laser, and a broad peak around 1 MHz is identified as the servo-bump of the reference laser PDH lock.

locked laser exhibits several frequency noise spikes in the range of 100–700 Hz, which we mainly attribute to the acoustic noise: vibrations of the experimental setup for the coupling between the laser chip and the Si_3N_4 photonic chip. We confirmed this by mechanical noise measurements with an accelerometer. With vibration cancellation and packaging, we expect an improvement of the noise level at frequency offsets <1 kHz and suppression of the mechanical chip modes at 1.5 kHz and 4.67 kHz for the 2.45 GHz devices. At high offset frequencies, we observe that the direct

heterodyne measurement of the noise is either limited by photon shot noise above 1 MHz (rising with f^2), the noise of the reference lasers or the PDH locking bandwidth. The difference in sensitivity between the different resonators towards the acoustic noise is related to the different chip size and clamping forces as well as the gap distance between the laser diode and the chip at the optimal phase setting. For the 2.45-GHz-FSR resonator, we observe additional noise peaks at 1.5 kHz, which we attribute to the technical noise from a vacuum pump placed in the lab, and at 5 kHz, which we attribute to the excitation of mechanical contour modes of the Si_3N_4 chip (20×20 mm size). We confirmed our assumption with FEM simulation and depict normalized displacement and frequency in the inset to Fig. 6. As shown in Fig. 5 of the SI loaded cavity linewidths are approximately the same across all resonances of the particular Si_3N_4device inside the DFB tuning range 1555-1556.5 nm. Thus, the noise suppression factor due to the laser self-injection locking to different cavity resonance would not vary by more than a factor of 4. Thus, we can choose any microresonator resonance inside DFB current tuning range for laser self-injection locking with proper feedback phase adjustment to obtain a low noise performance at different wavelengths. In the future, by reducing the Si_3N_4 chip size and the resonator footprint by using spiral or meander-shaped resonators, we can eliminate most of these noise peaks while simultaneously decreasing the linewidth due to the favorable scaling of TRN noise with the cavity mode volume.

Fig. 7 presents frequency noise measurement for all reference lasers used in our work. For frequency noise measurements of SIL DFB (Fig. 2 of the main manuscript) we used as a reference laser Toptica CTL PDH-locked to a crystalline MgF_2 resonator. The frequency noise of the PDH locked Toptica CTL is plotted as red line in Fig. 7. It was measured by heterodyne beat spectroscopy with the MenloSystems ORNS (Fig. 7 black trace). The phase noise was provided by the manufacturer. To measure tuning linearity and range for Fig. 3 of the main manuscript we used a free-running Toptica CTL (no PDH lock) and plot its frequency noise as pink line in Fig. 7 for comparison. Excess phase noise of the Toptica laser is observed mostly at frequncies below 10 kHz and effectively removed by the PDH lock.

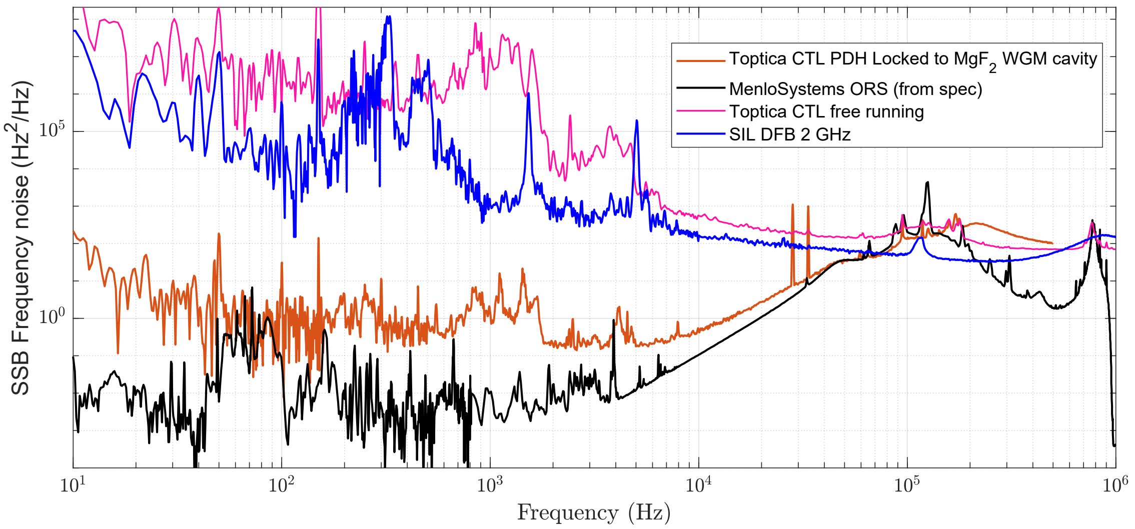

Figure 7. **Frequency noise of the reference lasers.** (black) MenloSystems ORNS data from specs; (red) Toptica CTL locked to crystalline WGM resonator using PDH technique used as a reference for Fig. 2 of the main Manuscript; (pink) free-running Toptica CTL used as a reference for Fig. 3(d) of the main Manuscript; (blue) SIL DFB 2 GHz FSR.

Optical cross correlation measurement of laser phase noise

At high offset frequencies, the sensitivity of laser frequency noise measurement by homodyne or heterodyne beat spectroscopy can be limited due to the white phase noise detection floor of photon shot noise that is scaled by the factor f^2 in the phase noise to frequency noise conversion. The second method by which we measure the frequency noise of the SIL laser is cross-correlation spectroscopy. Optical cross correlation is the technique of choice for the characterization of lasers [13] and RF oscillators [14] with the highest coherence. We use free-running Toptica CTL lasers as reference lasers, and the two heterodyne beat notes are recorded using balanced photoreceivers (New Focus 1817). The lasers and photodiodes use independent power supplies to minimize correlated noise sources. The optical setup is depicted in Fig. 3 of the main manuscript. The reference lasers are both red-detuned around 20 MHz with

respect to the self-injection locked laser and the beat signals are sampled directly on a fast sampling digitizer with 50 Msps for 100 s. The phase of the heterodyne beat signals are extracted using Hilbert's transform

$$\Phi_{\text{meas}}(t) = \arg \mathcal{H}\left\{U(t)\right\}, \tag{6}$$

where $U(t)$ denotes the measured voltage of the heterodyne beat note. The carrier frequency of the beat notes is subtracted by subtracting a linear fit of $\Phi_{\text{meas}}(t)$. The single-sided power spectral densities (PSD) of the two beatnotes and the cross power spectral density (CPSD) are calculated using MATLAB. At higher offset frequencies, the traces are segmented prior to Fourier transform and averaged after Fourier transform, and the results are depicted in Fig. 8. The segment lengths are as follows: 100 ms, 33 ms, 10 ms, 3.3 ms, 1 ms, 330 μs, 100 μs, 33 μs, 10 μs and segmentation is performed with 50% overlap between neighbouring segments. PSD and cross-PSD is calculated by Fourier-transform using Welch's method. The true phase noise of the SIL laser is revealed after averaging of the phase noise cross-PSD over many time intervals m according to $S_{\phi\phi}(f) = \Re\langle S_{AB}(f)\rangle_m + \mathcal{O}\left(m^{-1/2}\right)$, where the subscripts A, B denote the two heterodyne beat notes and S_{AB} their cross-PSD. The biggest drawback of the cross-correlation measurement comes from the requirement of exceedingly long averaging times for the determination of the noise at low offset frequencies. All the relevant information on the device phase noise is retained in the real part of the cross-power spectral density $\Re\langle S_{AB}\rangle$, and the correlated phase noise of the SIL laser appears as a positive correlation only [15]. By analyzing its sign, we can identify the low frequency cut-off below which averaged cross power spectral density is dominated by residual random (anti-)correlation of the ECDL noises. We find that below 5 kHz the unlocked ECDL noise becomes too large and completely dominates the SIL laser noise in our measurement and the measurement time of 100 s is insufficient. The frequency flicker noise floor of both measurement methods agrees well in the region between 10 kHz and 1 MHz, where the cross correlation method reveals several noise peaks of the reference laser. The real part of the optical cross correlation signal contains the common-mode noise of the two beatnotes, which is the phase noise of the self-injection locked laser and the suppression of uncorrelated noises such as reference laser noise and photon shot noise reduce as $\sqrt{N}$, where N is the number of averages. We plot both the positive and the negative part of the CPSD to inspect for possible anti-correlated noise sources that would give a false and lower estimate of the phase noise [15]. From this, we can conclude that optical cross correlation provides a precise estimation of the SIL laser phase noise for offset frequencies between 5 kHz and 5 MHz. The lower limit is given by insufficient averaging to remove the excess phase noise and spurious anti-correlations of the reference lasers. The upper limit is given by the carrier frequency of the beat notes and the Nyquist sampling limit of the analog-to-digital conversion.

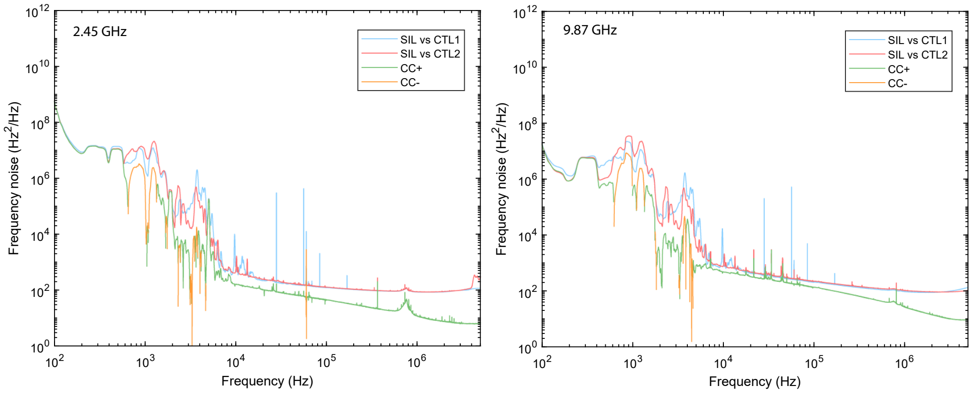

Figure 8. **Optical cross correlation measurement.** Single-sided PSD and CPSD for optical cross-correlation phase noise measurement. The single-sided PSD of the two beatnotes for optical cross-correlation is depicted in blue (SIL vs CTL1) and red(SIL vs CTL2). The positive part of the real part of the CPSD (common-mode noise) is depicted in green (CC+), and the negative part (spurious anti-correlation) is depicted in orange (CC-). The left panel shows the results for the 2.45 GHz device and the right panel shows the results for the 9.87 GHz device.

Phase noise measurement of frequency-modulated laser

The phase of a rapidly frequency-modulated laser, such as a FMCW laser source, is defined as the time integral of over the target triangular ramp frequency-modulation.

$$\Phi(t) = \Phi(0) + \omega_0 \cdot t + \int_0^t \Delta\omega(t')dt' \tag{7}$$

For a direct comparison of the laser phase noise in the CW and FMCW operation modes, we can extract the modulated laser phase from the heterodyne beat spectroscopy experiment depicted in Fig. 3 of the main manuscript using Hilbert's transform (eq. 6) and compare with the target modulation. A free-running Toptica CTL laser was used as a reference in the experiment. Residual intensity noise of the lasers is suppressed by 10 MHz high pass filtering of the beat note prior to phase extraction. This does not influence the phase noise extraction because the lowest carrier frequencies of the beat note are around 500 MHz. The top row of Fig. 9 shows the phase deviation $\Delta\Phi$ between the measured phase and the phase of an ideal triangular frequency modulation, which is extracted from our fit of the frequency-modulation after subtraction of a linear polynomial to eliminate the constant phase and beat note carrier frequency offsets. For higher modulation speeds the phase deviation is usually confined around one of the two turning points, which we attribute to a decrease of the phase noise reduction close to the edge of the self-injection locking bandwidth. The phase noise power spectral densities are depicted in the lower row of Fig. 9. In each case, the measurement duration corresponds to between seven and ten periods of the triangular chirp. At low offset frequencies, the phase

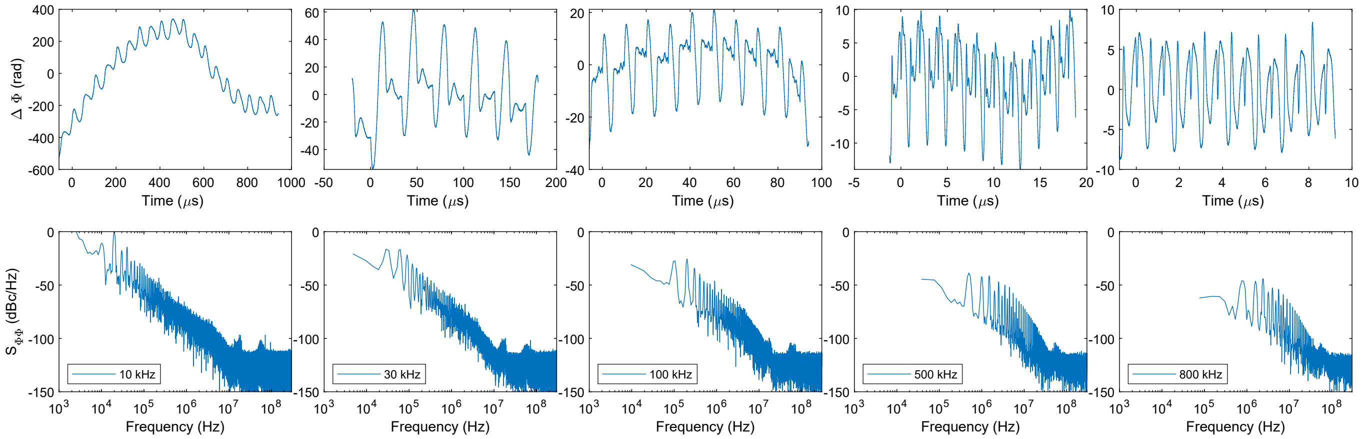

Figure 9. **Phase noise of triangular frequency-modulated laser**. The top row depicts the phase difference between the measured heterodyne beat note and the ideal phase curve of a triangular frequency-modulated laser. The bottom row depicts the single-sided power spectral density of the phase deviation. The legend indicates the repetition frequency of the triangular ramp chirps.

noise PSD is dominated by the residual nonlinearities of the odd and even harmonics of the repetition frequency of the triangular chirp. These could be further reduced by pre-distortion [16] of the driving signal or by phase locking to an imbalanced Mach-Zehnder Interferometer [17]. For chirp repetition frequency at 10 kHz, we find phase noise of around -80 dBc/Hz for offset frequencies around 1 MHz (maximum value -71 dBc/Hz at 970 kHz -93 dBc/Hz at 983 kHz), which corresponds to 10^4 Hz^2/Hz and is within a factor of ten from the CW case for self-injection locking to the 190.7 GHz microresonator. At 30 kHz modulation and 1 MHz offset frequency, we start to see stronger overtones in the phase noise of the odd harmonics (33rd & 35th). Aside from the overtones, a baseline of -80 dBc/Hz is observed here. The closest overtone has a maximum values of -62 dBc/Hz at 991 kHz.

Intensity noise measurement

The relative intensity noise (RIN) is important to characterize the laser performance for numerous applications, especially in optical communication systems. The RIN is measured with a DC-coupled photodiode for the case of the 9.87 GHz microresonator (see Figure 10). A large DC block capacitor (100 μF) with frequency cut-off below 1 Hz is inserted to separately measure the DC photocurrent. The noise fluctuation is measured with the same electrical spectrum analyser that was used for the frequency noise measurement. Compared with the RIN of the laser in free-running condition, the injection-locked laser exhibits a noticeable RIN increase at frequencies below 10 kHz, which is caused by the vibrations of mechanical stages that constitute the coupling setup between the laser chip and the Si_3N_4 photonic chip. With better vibration cancellation or compact and rigid component packaging, the excess frequency noise and RIN deterioration at low offset frequencies can be avoided.

No relaxation oscillations peaks were observed in our RIN measurements for the free-running and self-injection locked DFB. According to the simulations presented above, a relaxation oscillation frequency of the DFB laser should be above 10 GHz both in the free-running and in the SIL state.

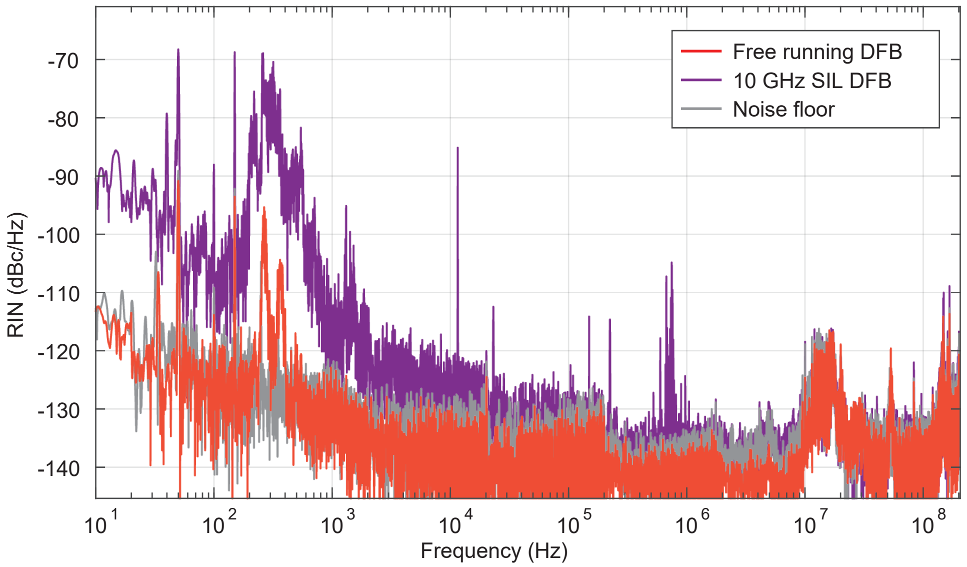

Figure 10. **Laser RIN measurement.** DFB laser is free-running (red) or self-injection locked to the 9.78 GHz Si_3N_4 microresonator (violet). We mainly attribute observed excess noise at 100–700 Hz to vibrations of the experimental setup.

Free running DFB characterization

We also performed basic characterization measurements of the free running DFB laser without the Si_3N_4chip. Figure 11 (a) shows the free-space optical power as function of the DFB laser diode current. Figure 11 (b) presents the corresponding optical spectra of the DFB laser diode at different driving currents obtained by approaching a lensed fiber to the laser diode directly.

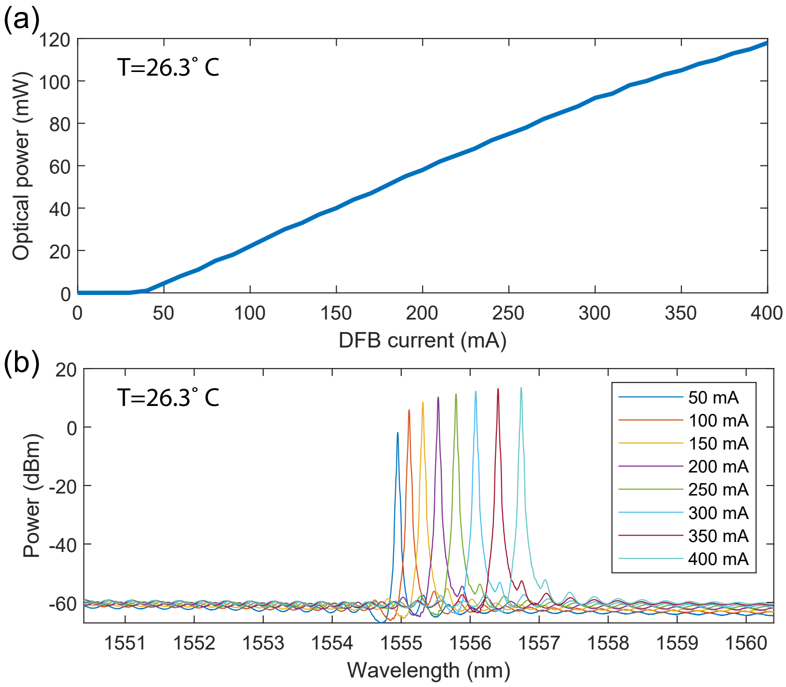

Figure 11. **Free running DFB characterization.** a) Laser diode free-space optical power vs. diode current. b) Free-running DFB optical spectra at different driving currents.

We also compare the frequency tuning of the bare DFB laser and the hybrid integrated laser and estimate the corresponding linearity of tuning without active linearization and predistortion using the same experimental setup and analysis method as in Fig. 3 of the main manuscript. We drive the laser diode with a current of 155 mA and a 3.5 mA triangular chirp at 1 kHz and 10 kHz repetition rates. Higher current modulation frequencies are limited by the bandwidth of our low noise current controller (ILX LDX-3620B). We choose a 3.5 mA current ramp amplitude corresponding to a 1.2 GHz optical frequency excursion similar to the SIL tuning case. Fig. 12 shows the time-frequency spectrogram of the heterodyne beat-notes with a fixed frequency reference laser (Toptica CTL) for 1 kHz and 10 kHz chirp repetition rates. The bottom row reveals RMS nonlinearities 30 MHz for 1 kHz tuning rate and 120 MHz for 10 kHz. Such nonlinearities are two orders of magnitude higher than the values measured in self-injection locked laser regime with piezoactuator voltage tuning.

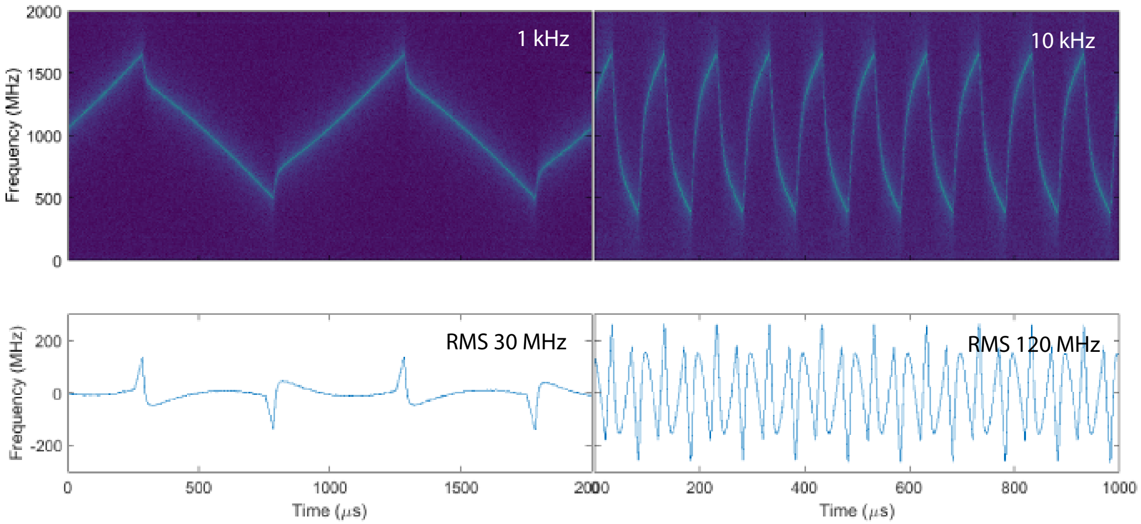

Figure 12. **Free running DFB frequency tuning linearity using current tuning.** Time-frequency spectrogram of the heterodyne beatnotes for 1 kHz and 10 kHz triangular chirp frequencies. Bottom row: Residual of least-squares fitting of the time-frequency traces with symmetric triangular chirp pattern.

Frequency noise and tuning comparison between different compact lasers

Fig. 13 compares frequency noise of hybrid and heterogeneous integrated lasers reported in recent literature. The material systems used include glass (Morton Photonics [18]), high-confinement Si_3N_4 (Columbia [19], Tsinghua University [20]), weak-confinement Si_3N_4 (UCSB [21] & [22], University of Twente [23]). The list includes both heterogeneous- (UCSB [22]) and hybrid-integrated laser systems based on low-confinement Si_3N_4 ring resonators [21]. The lowest reported value of frequency noise in similar systems was achieved in packaged systems with a InP DFB laser self-injection locked to a bulk crystalline MgF_2 [24].

Table II presents a comparison of different tunable laser systems in terms of the frequency tuning range, tuning rate, linearity, optical output power, frequency white noise floor. A wide array of recent systems using a range of optical integration technologies ranging from monolithic InP digital-supermode distributed Bragg-reflector lasers (DS-DBR) and distributed-feedback (DFB) lasers (including active linearization and external feedback systems) to MEMS-tunable vertical cavity surface emitting lasers (VCSEL) to commercial ECDL and fibre lasers is chosen. The list is non-exhaustive given the large number of tunable laser systems and applications. Recent examples of hybrid integrated lasers using low confinement Si_3N_4 are added to the list despite not having been integrated with fast non-thermal tuning so far.

LiDAR data analysis

The collected data were processed in order to obtain the location of the objects comprising the studied scene. First, the padding of oscillogram was carried out to align the zero point due to different time delays between the oscilloscope

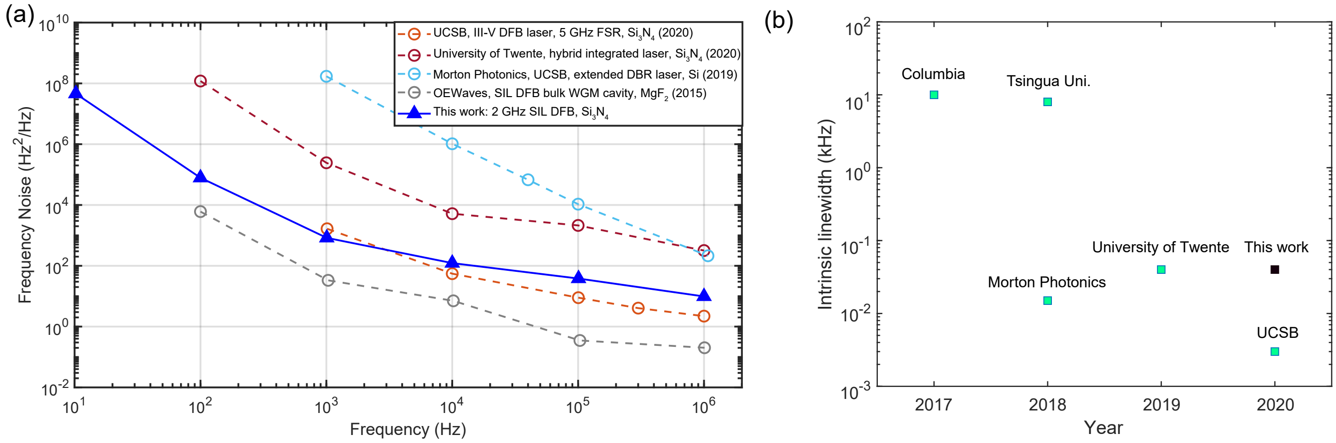

Figure 13. **Frequency noise of different integrated lasers.** a) Frequency noise of several contemporary hybrid and heterodyne integrated lasers. Citations are found in the SI text. b) Intrinsic linewidth of different lasers reported by universities or companies.

trigger signal and LiDAR signal path. The second step was to perform short-time Fourier transform (STFT) of the oscillograms from the target and the reference MZI. The Blackman-Harris window function was chosen for STFT with the window size set to half of the laser frequency chirping period. Next, time-frequency spectrograms were analysed, and the maximal values of each timeslice were identified for the subsequent conversion of the corresponding frequency points into the distance units. An alternative approach consisted of the application of a Gaussian fitting procedure for searching the maximal spectrum values. After that, the noise filtering stage followed that cut off the identified peaks lower than a given value of 10 dB that is attributed to noise or low reflection. Next, the galvo-mirror angular coordinates linearization was applied to reduce the digitalization noise smoothing the beam scanning pattern to the linear ramp shape. Finally, a three-dimensional spatial distribution of the detected points was plotted with distance-based coloring. To achieve a greater quality of image, additional filtering or N-points-averaging can be performed for a point cloud to reduce a discretization error which is due to 1) a LiDAR resolution of 12.5 cm and 2) the distance ambiguity related to the laser frequency Doppler shift due to a fast rotation of galvo mirrors (60 Hz in our case). No additional filtering has been applied to an image presented in Fig. 4 of the main manuscript. We attach an interactive Python script to the Zenodo data and code repository for LiDAR data processing with a detailed step-by-step descriptions.

Mode-cancellation schemes for actuation bandwidth extension

Contour modes of an integrated MEMS-photonic chip have their fundamental frequency defined by its in-plane dimensions, and the operational modes can be broadly categorized into flexural and bulk modes [39]. At first, we develop a difference-actuation scheme. In this scheme, besides the AlN actuator above the microresonator, an additional AlN actuator with the same geometry is fabricated adjacent to the microresonator but with no Si_3N_4 microring resonator below it. During actuation, the two actuators are driven by the same frequency with an identical amplitude but different phases to cancel the actuation of the photonic chip mechanical modes. As a result, while the stress-optical effect exerted on the microresonator is the same, the detrimental flexural modes arising due to transverse standing waves get effectively suppressed. Fig. 3 (e) in the main manuscript shows the measured response of the conventional single-actuator configuration and the difference-actuation configuration. The comparison clearly shows that the latter effectively reduces the amplitudes of the mechanical modes below 1 MHz (green trace), mainly cancelling the flexural modes due to far-field destructive interference. It is clearly observed that the bulk mechanical mode at 967 kHz does not get affected by this scheme. We use the finite-element method to compute the contour modes' profiles of an actual photonic chip with the size of 4.96 mm x 4.96 mm. In insets of Fig. 3 (e) we show three of these eigenmodes. The eigenmodes at 225 kHz and 490 kHz are flexural modes, whereas the one at 967 kHz is a bulk mode. The actuation voltage derived from a vectorial network analyser (VNA) is applied on the two actuators in an anti-phase fashion, and a laser is frequency-tuned to sit on the side of resonance. The frequency modulation due to the actuation is converted to the intensity modulation of the transmitted laser light that is received by a fast photodetector.

Next, we implement apodized shape engineering on the photonic chips. Many mechanical modes of relatively low

Author	System	Frequency white noise floor	Tuning speed, $f_{mod} * B/\text{Hz}^2$	Tuning rate (f_{mod})	Tuning range (B)	Linearity	Optical output power (mW)
S. Yoo et al.[25]	DS-DBR	5 MHz	1.54E+16	1.23 MHz	12.5 GHz	Low, Wavelength switching	6
N. Nunoya et al.[26]	TDA-DFB	n/a	3.75E+15	5 kHz	0.75 THz	Low, Wavelength switching	20
Y. Fan et al.[27]	Si_3N_4Vernier filter	40 Hz	n/a	thermal	8.7 THz	n/a	23
M. A. Tran et al.[28]	Heterogeneous Si_3N_4/InP	220 Hz	n/a	thermal	30 GHz (continuous)	Low	3.5
W. Jin et al.[21]	Heterogeneous Si_3N_4/InP	1.2 Hz	n/a	n/a	n/a	n/a	3.5
C.V. Poulton et al.[29]	InP DFB	n/a	6.4E+12	100 Hz	64 GHz	High	1
N. Satyan et al.[30]	InP DFB w. external feedback	1 MHz	1E+14	1 kHz	100 GHz	High (OPLL)	40
X. Zhang et al.[31]	VCSEL	n/a	7.75E+14	5 kHz	155 GHz	High (ILC pre - distortion)	n/a
Measured EPFL	Toptica CTL	90 Hz	2.5E+14	100 Hz	2.5 THz	Low	30
Measured EPFL	Koheras Adjustik E15	<7 Hz	2E+13	20 kHz	1 GHz (piezo)	Low	1.5
Datasheet[32]	Lumentum NPRO 126	<5 kHz	9E+11	30 kHz	30 MHz	n/a	>100
Datasheet[33]	Insight Photonics	1.7 GHz	7E+18	400 kHz	18 THz	High	<20
M. Okano et al.[34]	VCSEL	<1 MHz	1.1E+17	10 kHz	11 THz	High (k-point sampling)	1
DiLazaro et al.[35]	12 stitched DFBs	3 MHz	1.8E+15	330 Hz	5.56 THz	High (with linearization)	12.7
Latkowski et al.[36]	intracavity MZI	363 kHz	n/a	n/a	9.6 THz	n/a	3
Ward et al.[37]	DS-DBR	400 kHz	n/a	n/a	n/a	n/a	25
Wei et al.[38]	Inj. locking to EOM sideband	3 kHz	n/a	2.5E+12	15 GHz	High	15
Morton et al.[18]	RSOA+FBG	15 Hz	n/a	n/a	n/a	Low	100
Stern et al.[19]	RSOA+Si_3N_4	13 kHz	n/a	n/a	n/a	n/a	1.7
Li et al.[20]	FP+Si_3N_4	8 kHz	n/a	n/a	n/a	Wavelength switching	5
This work	SIL DFB	25 Hz	1.6E+15	800 kHz	2 GHz	High	1.5

Table II. Performance comparison of tunable laser systems.

resonance frequencies are flexural modes whose vibrations are caused by transverse standing waves [40]. The bulk mechanical modes, whose vibrations are caused by longitudinal standing waves, can be eliminated by judiciously shaping the geometry of the photonic chips [41, 42]. The apodization was performed by dicing the released chip, see Fig. 14. We observed a reduction in the number of bulk mechanical modes in an apodized photonic chip. We repeat the actuation response measurement, and in the result that is presented in Fig. 3 (e) of the main manuscript, it is shown that the mechanical resonances below $\sim$ 1.69 MHz are significantly suppressed (red trace). We confirm first mechanical mode of the apodized chip at 1.69 MHz with FEM simulations. We further flatten the actuation response by attaching the apodized chip on a piece of carbon tape and then differentially driving the actuators, as explained before. In this way, both the flexural and the bulk mechanical modes are damped up to the first HBAR mode at

17 MHz as shown in Fig. 15 (a). It is also observed that even without using the difference-actuation scheme, all the mechanical resonances of the apodized chip are significantly suppressed till the first HBAR mode, but the difference-actuation scheme on an apodized chip placed over a carbon tape limits the fluctuation of the actuation response within 1 dB, giving the best flattened result as depicted in Fig. 15 (b). This actuation response can significantly improve the linear chirping performance of the system as a LiDAR engine.

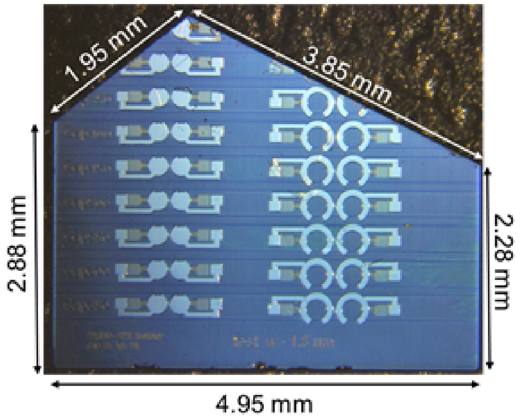

Figure 14. **Photo of the apodized chip.**

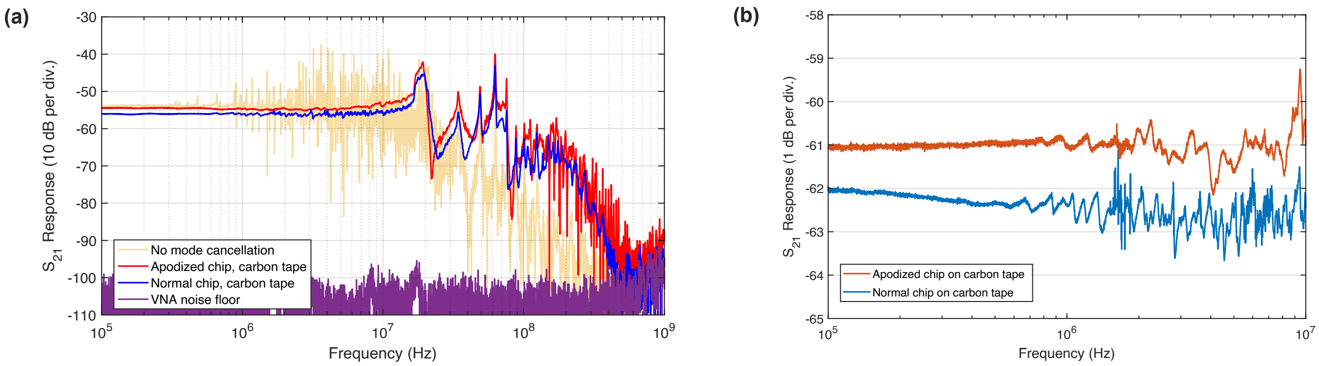

Figure 15. **S_{21} response showing mechanical resonances of the photonic chip**. (a) Full spectrum showing suppression of mechanical modes using apodized chip and normal square chip placed over a piece of carbon tape. (b) Apodized chip shows response fluctuation within 1 dB due to effective suppression of bulk mechanical modes as compared to a normal chip.

Actuation bandwidth requirements for targeted chirp linearity

We present the analysis of piezoelectric actuator bandwidth requirements to achieve target linearity for a triangular chirp. In our simplified model we consider an actuator as a low pass RC filter. Varying the cutoff frequency of the filter we simulated filtering of perfect triangular signal. Figure 16(a) shows the ideal triangular signal at 100 kHz frequency and simulated triangular signals with lowpass RC filtering applied for different filter cutoff frequencies. Figure 16(b) shows RMS nonlinearity vs RC-filter cutoff frequency for different analysis interval: 100% of ramp-up (blue), central 90% of ramp (red), central 80% of ramp (orange). 10 MHz actuator bandwidth provides 100kHz tuning rate in linear chirp with 0.03% RMS nonlinearity (normalized to the tuning range) over the full tuning range.

---

* These authors contributed equally

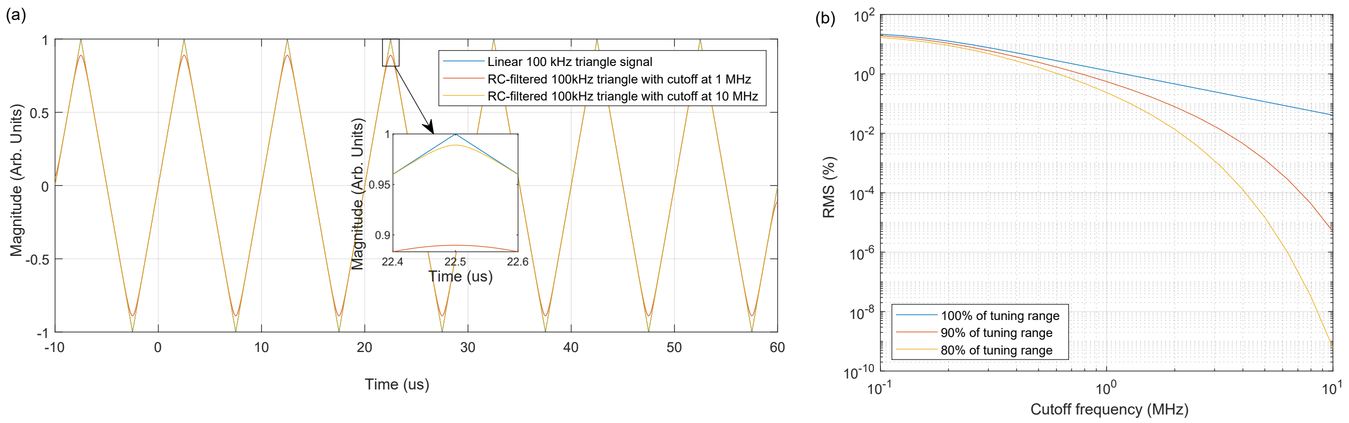

Figure 16. **Lowpass RC filtering of triangular signal with different cutoff frequency**. Perfect triangular signal (blue) with 100 kHz frequency, triangular signal with applied lowpass filter function with 1 MHz cutoff frequency (red) and 10 MHz (orange). Inset: zoom into the turning point. (b) RMS nonlinearity vs RC filter cutoff frequency for different analysis interval: 100% of ramp (blue), central 90% of ramp (red), central 80% (orange).

† bhave@purdue.edu
‡ tobias.kippenberg@epfl.ch

[1] G. Agrawal, Line narrowing in a single-mode injection laser due to external optical feedback, IEEE Journal of Quantum Electronics **20**, 468 (1984).
[2] J. Dellunde, M. Torrent, J. Sancho, and M. San Miguel, Frequency dynamics of gain-switched injection-locked semiconductor lasers, IEEE journal of quantum electronics **33**, 1537 (1997).
[3] J. Ohtsubo, *Semiconductor lasers: stability, instability and chaos*, Vol. 111 (Springer, 2012).
[4] D. Welford and S. Alexander, Magnitude and phase characteristics of frequency modulation in directly modulated GaAlAs semiconductor diode lasers, Journal of Lightwave Technology **3**, 1092 (1985).
[5] N. Kondratiev, V. Lobanov, A. Cherenkov, A. Voloshin, N. Pavlov, S. Koptyaev, and M. Gorodetsky, Self-injection locking of a laser diode to a high-Q WGM microresonator, Optics Express **25**, 28167 (2017).
[6] P. Laurent, A. Clairon, and C. Breant, Frequency noise analysis of optically self-locked diode lasers, IEEE Journal of Quantum Electronics **25**, 1131 (1989).
[7] M. H. P. Pfeiffer, C. Herkommer, J. Liu, T. Morais, M. Zervas, M. Geiselmann, and T. J. Kippenberg, Photonic damascene process for low-loss, high-confinement silicon nitride waveguides, IEEE Journal of selected topics in quantum electronics **24**, 1 (2018).
[8] J. Liu, E. Lucas, A. S. Raja, J. He, J. Riemensberger, R. N. Wang, M. Karpov, H. Guo, R. Bouchand, and T. J. Kippenberg, Photonic microwave generation in the x-and k-band using integrated soliton microcombs, Nature Photonics , 1 (2020).
[9] M. H. Pfeiffer, J. Liu, A. S. Raja, T. Morais, B. Ghadiani, and T. J. Kippenberg, Ultra-smooth silicon nitride waveguides based on the damascene reflow process: fabrication and loss origins, Optica **5**, 884 (2018).
[10] J. Liu, A. S. Raja, M. H. Pfeiffer, C. Herkommer, H. Guo, M. Zervas, M. Geiselmann, and T. J. Kippenberg, Double inverse nanotapers for efficient light coupling to integrated photonic devices, Optics letters **43**, 3200 (2018).
[11] P. Del'Haye, O. Arcizet, M. L. Gorodetsky, R. Holzwarth, and T. J. Kippenberg, Frequency comb assisted diode laser spectroscopy for measurement of microcavity dispersion, Nature Photonics **3**, 529 (2009).
[12] R. R. Galiev, N. M. Kondratiev, V. E. Lobanov, A. B. Matsko, and I. A. Bilenko, Optimization of laser stabilization via self-injection locking to a whispering-gallery-mode microresonator, Phys. Rev. Applied **14**, 014036 (2020).
[13] X. Xie, R. Bouchand, D. Nicolodi, M. Lours, C. Alexandre, and Y. Le Coq, Phase noise characterization of sub-hertz linewidth lasers via digital cross correlation, Optics letters **42**, 1217 (2017).
[14] E. Rubiola and V. Giordano, Correlation-based phase noise measurements, Review of Scientific Instruments **71**, 3085 (2000).
[15] Y. Gruson, A. Rus, U. L. Rohde, A. Roth, and E. Rubiola, Artifacts and errors in cross-spectrum phase noise measurements, Metrologia **57**, 055010 (2020).
[16] P. Feneyrou, L. Leviandier, J. Minet, G. Pillet, A. Martin, D. Dolfi, J.-P. Schlotterbeck, P. Rondeau, X. Lacondemine, A. Rieu, *et al.*, Frequency-modulated multifunction lidar for anemometry, range finding, and velocimetry—2. experimental results, Applied optics **56**, 9676 (2017).
[17] P. A. Roos, R. R. Reibel, T. Berg, B. Kaylor, Z. W. Barber, and W. R. Babbitt, Ultrabroadband optical chirp linearization for precision metrology applications, Optics letters **34**, 3692 (2009).
[18] P. A. Morton and M. J. Morton, High-power, ultra-low noise hybrid lasers for microwave photonics and optical sensing, Journal of Lightwave Technology **36**, 5048 (2018).
[19] B. Stern, X. Ji, A. Dutt, and M. Lipson, Compact narrow-linewidth integrated laser based on a low-loss silicon nitride ring resonator, Optics letters **42**, 4541 (2017).

[20] Y. Li, Y. Zhang, H. Chen, S. Yang, and M. Chen, Tunable self-injected fabry–perot laser diode coupled to an external high-q si_3n_4/sio_2 microring resonator, Journal of Lightwave Technology **36**, 3269 (2018).
[21] W. Jin, Q.-F. Yang, L. Chang, B. Shen, H. Wang, M. A. Leal, L. Wu, M. Gao, A. Feshali, M. Paniccia, *et al.*, Hertz-linewidth semiconductor lasers using cmos-ready ultra-high-q microresonators, Nature Photonics **15**, 346 (2021).
[22] C. Xiang, P. A. Morton, and J. E. Bowers, Ultra-narrow linewidth laser based on a semiconductor gain chip and extended Si_3 N_4 bragg grating, Optics letters **44**, 3825 (2019).
[23] K.-J. Boller, A. van Rees, Y. Fan, J. Mak, R. E. Lammerink, C. A. Franken, P. J. van der Slot, D. A. Marpaung, C. Fallnich, J. P. Epping, *et al.*, Hybrid integrated semiconductor lasers with silicon nitride feedback circuits, in *Photonics*, Vol. 7 (Multidisciplinary Digital Publishing Institute, 2020) p. 4.
[24] W. Liang, V. Ilchenko, D. Eliyahu, A. Savchenkov, A. Matsko, D. Seidel, and L. Maleki, Ultralow noise miniature external cavity semiconductor laser, Nature communications **6**, 1 (2015).
[25] S. Yoo, J. K. Lee, and K. Kim, Suppression of thermal wavelength drift in widely tunable ds-dbr laser for fast channel-to-channel switching, Opt. Express **25**, 30406 (2017).
[26] N. Nunoya, H. Ishii, Y. Kawaguchi, R. Iga, T. Sato, N. Fujiwara, and H. Oohashi, Tunable distributed amplification (tda-) dfb laser with asymmetric structure, IEEE Journal of Selected Topics in Quantum Electronics **17**, 1505 (2011).
[27] Y. Fan, A. van Rees, P. J. Van der Slot, J. Mak, R. M. Oldenbeuving, M. Hoekman, D. Geskus, C. G. Roeloffzen, and K.-J. Boller, Hybrid integrated InP-Si_3 N_4 diode laser with a 40-hz intrinsic linewidth, Optics express **28**, 21713 (2020).
[28] M. A. Tran, D. Huang, J. Guo, T. Komljenovic, P. A. Morton, and J. E. Bowers, Ring-resonator based widely-tunable narrow-linewidth si/inp integrated lasers, IEEE Journal of Selected Topics in Quantum Electronics **26**, 1 (2020).
[29] C. V. Poulton, A. Yaacobi, D. B. Cole, M. J. Byrd, M. Raval, D. Vermeulen, and M. R. Watts, Coherent solid-state lidar with silicon photonic optical phased arrays, Opt. Lett. **42**, 4091 (2017).
[30] N. Satyan, A. Vasilyev, G. Rakuljic, V. Leyva, and A. Yariv, Precise control of broadband frequency chirps using opto-electronic feedback, Opt. Express **17**, 15991 (2009).
[31] X. Zhang, J. Pouls, and M. C. Wu, Laser frequency sweep linearization by iterative learning pre-distortion for fmcw lidar, Opt. Express **27**, 9965 (2019).
[32] Lumentum, Lumentum npro125/126, https://resource.lumentum.com/s3fs-public/technical-library-items/npro125126-ds-cl-ae.pdf?null (2021).
[33] Insight, Insight akinetic swept laser, https://www.sweptlaser.com/akinetic-technology (2021).
[34] M. Okano and C. Chong, Swept source lidar: simultaneous fmcw ranging and nonmechanical beam steering with a wideband swept source, Opt. Express **28**, 23898 (2020).
[35] T. DiLazaro and G. Nehmetallah, Large-volume, low-cost, high-precision fmcw tomography using stitched dfbs, Opt. Express **26**, 2891 (2018).
[36] S. Latkowski, A. Hänsel, N. Bhattacharya, T. de Vries, L. Augustin, K. Williams, M. Smit, and E. Bente, Novel widely tunable monolithically integrated laser source, IEEE Photonics Journal **7**, 1 (2015).
[37] A. Ward, D. Robbins, G. Busico, E. Barton, L. Ponnampalam, J. Duck, N. Whitbread, P. Williams, D. Reid, A. Carter, and M. Wale, Widely tunable ds-dbr laser with monolithically integrated soa: design and performance, IEEE Journal of Selected Topics in Quantum Electronics **11**, 149 (2005).
[38] F. Wei, B. Lu, J. Wang, D. Xu, Z. Pan, D. Chen, H. Cai, and R. Qu, Precision and broadband frequency swept laser source based on high-order modulation-sideband injection-locking, Opt. Express **23**, 4970 (2015).
[39] G. Piazza, P. J. Stephanou, and A. P. Pisano, Piezoelectric aluminum nitride vibrating contour-mode mems resonators, Journal of Microelectromechanical systems **15**, 1406 (2006).
[40] G. Wu, J. Xu, E. J. Ng, and W. Chen, Mems resonators for frequency reference and timing applications, Journal of Microelectromechanical Systems **29**, 1137 (2020).
[41] R. C. Ruby, Piezoelectric resonator structures and electrical filters (2009), uS Patent 7,629,865.
[42] D. Burak, J. Choy, and C. Feng, Acoustic resonator device with structures having different apodized shapes (2017), uS Patent 9,571,063.
